# Protective Effects of Dioscin and Diosgenin on Plateau Hyperuricemia by Attenuating Renal Inflammation via EPHX2

**DOI:** 10.3390/ijms252413399

**Published:** 2024-12-13

**Authors:** Wanyun Dang, Fangyang Li, Rong Gao, Cheng Zhang, Hongbo Cheng, Zhenhui Wu, Tingyu Yang, Jinchao Pan, Xianglin Tang, Yue Gao

**Affiliations:** 1 School of Pharmacy, Guangdong Pharmaceutical University, Guangzhou 510006, China; wanyundang@163.com (W.D.); lifangyang7012@163.com (F.L.); gaoronger198@163.com (R.G.); cadendengdengdeng@163.com (C.Z.); chb0000@163.com (H.C.); 2 Beijing Institute of Radiation Medicine, Beijing 100859, China; wzh77580@163.com (Z.W.); 15254555178@163.com (T.Y.); llpp919227@163.com (J.P.); 3 School of Chinese Materia Medica, Tianjin University of Traditional Chinese Medicine, Tianjin 301617, China; 4 Faculty of Environment and Life, Beijing University of Technology, Beijing 100124, China

**Keywords:** plateau hyperuricemia, dioscin, giosgenin, EPHX2, lipid metabolism, anti-inflammatory

## Abstract

Plateau hyperuricemia is a common disease in the plateau area, and the incidence is much higher than that in the plain area. Dioscin (DIO) and its active metabolite Diosgenin (DG) exert therapeutic effects on hyperuricemia through oxidative stress and inflammation. In this study, DIO and its active metabolite DG were taken as the research objects to explore their therapeutic effects on high-altitude hyperuricemia in rats. To evaluate the therapeutic effect of DIO on the rat model of high-altitude hyperuricemia, the evaluation indexes include blood biochemical indexes, renal histopathology, oil red O staining of the kidney, rat kidney index, and rat renal inflammatory factors. Transcriptomics was used to analyze the control group, model group, and drug-administered group to preliminarily explore the protective mechanism of DIO in rats with high-altitude hyperuricemia. An HK-2 high-altitude hyperuricemia cell injury model was established to verify the therapeutic mechanism of DIO in rats with high-altitude hyperuricemia. Western blot was used to detect the expression of related proteins in renal tissues and cell models. The results showed that DIO and its active metabolite DG regulate renal lipid metabolism through the EPHX2 gene, attenuate renal inflammatory reaction, and then promote the excretion of uric acid and reduce its reabsorption, which ultimately achieves the effect of treating plateau hyperuricemia.

## 1. Introduction

A plateau is defined as an area located 3000 m above sea level. The unique geographic environment of the plateau, characterized by low pressure, low oxygen levels, strong sunlight, and low temperatures, triggers a series of acute and chronic diseases in its residents [1]. The high incidence of hyperuricemia in the plateau region has seriously affected the quality of life of plateau residents [2]. Although a comprehensive study of the pathogenesis of plateau hyperuricemia has not been conducted, reports indicated that the high incidence of hyperuricemia in plateaus is associated with a high-purine dietary intake combined with a hypoxic environment [1]. The kidneys, as one of the main organs for uric acid excretion and also one of the most energy-consuming organs in the human body, mainly rely on fatty acid oxidation to supply energy. In the hypoxic environment of the plateau, the blood oxygen concentration decreases, which causes sympathetic nerve excitation, an increase in blood pressure of the body, and an acceleration of blood circulation. Meanwhile, the blood vessels in the kidneys contract and the blood flow decreases, resulting in intensified excretion and reabsorption functions of the kidneys and eventually leading to kidney cell damage [3]. Moreover, exposure to the plateau can cause oxidative stress and inflammatory responses in the kidneys [1]. Some studies have also found that the hypoxic environment on the plateau can disrupt the fatty acid metabolism in the kidneys of rats, causing lipid accumulation [4,5]. Lipid accumulation will trigger oxidative stress and inflammatory damage to the kidneys, which will further lead to kidney damage and affect its function of excreting uric acid [6]. To date, there are no specific drugs or efficacious therapies for plateau hyperuricemia, making it particularly important to investigate potential therapeutic agents and interventions.

Dioscin (DIO) is a spirosteroidal glycoside, which has a significant role in improving cardiovascular disease, lowering uric acid, reducing inflammation, acting as an antioxidant, and mediating metabolic disorders [7,8,9]. Some studies have shown that dioscin can regulate lipid metabolism in mice to exert a uric acid-lowering effect. It can also protect renal tubular epithelial cells by alleviating the inflammatory response by inhibiting the NF-κB signaling pathway [10]. Moreover, it can reduce the oxidative stress of renal tubular epithelial cells by regulating the GSK3β/Nrf2/HO-1 signaling pathway [11] and mitigate the inflammatory response to protect the kidneys by regulating the microRNA let-7i/TLR4/MyD88 signaling pathway [12]. In rats, dioscin can be metabolized into diosgenin (DG) [13]. Diosgenin has anti-cancer and anti-inflammatory properties and can also regulate lipid metabolism in the body [14]. Furthermore, existing studies have found that lipid accumulation and inflammatory damage will occur in the kidneys of hypoxic rats. In view of these findings, we hypothesized that dioscin and its active metabolite diosgenin can protect the kidneys through anti-inflammation and increase uric acid excretion. However, the efficacy and potential mechanisms of dioscin and its metabolite diosgenin in treating plateau hyperuricemia remain unknown.

Therefore, this study aims to evaluate whether dioscin and its metabolite diosgenin can protect the kidneys by regulating lipid metabolism and alleviating inflammatory damage to the kidneys, increase uric acid excretion, and thus serve as a method for treating plateau hyperuricemia.

## 2. Results

### 2.1. Dioscin Reduces the Kidney Index, UA, CREA, BUN, and TG in the SD Model Rats

**Figure 1:**
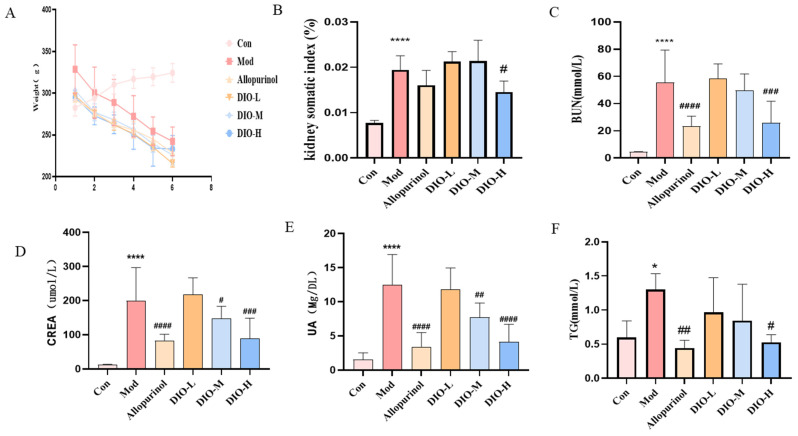
DIO improves renal indices and serum biochemical indices in rats. (**A**) Body weight. (**B**) Renal body mass index. (**C**) BUN. (**D**) CREA. (**E**) UA. (**F**) TG. **** *p* < 0.0001, * *p* < 0.05 vs. control group; ^####^ *p* < 0.0001, ^###^ *p* < 0.001, ^##^ *p* < 0.01, ^#^ *p* < 0.05 vs. model group. data are expressed as mean ± standard deviation (SD), *n* = 5. Con: Control group; Mod: model group; Allopurino: allopurinol group; DIO-L, M, H: Dio low-, medium-, and high-dose groups. Renal index = kidney weight (g)/body weight (g).

The results showed that compared with the rats in the Con group, the body weights of the rats decreased significantly after the hypobaric hypoxia experiment, but there was no significant difference between the drug-administered group and the model group (Figure 1A). In the Mod rats, the kidney index, UA (uric acid), CREA (creatinine), BUN (blood urea nitrogen), and TG (triglyceride) increased significantly (*p* < 0.0001, *p* < 0.05; Figure 1B–F). The kidney index, CREA, and BUN are crucial indicators representing the integrity of kidney function. The increase in these indicators in the model group indicated that kidney damage occurred in the rats of the model group. Compared with the Mod group, the kidney index in the high-dose group decreased significantly (*p* < 0.05; Figure 1B). The BUN in the high-dose group decreased significantly (*p* < 0.001; Figure 1C). The CREA in the medium- and high-dose groups decreased significantly (*p* < 0.05, *p* < 0.001; Figure 1D). The UA in the medium- and high-dose groups decreased significantly (*p* < 0.01, *p* < 0.0001; Figure 1E). The TG in the high-dose group decreased significantly (*p* < 0.05; Figure 1F). Overall, dioscin reduced the uric acid level in the model rats. Moreover, it can be seen from the kidney index, CREA, and BUN that dioscin improved kidney function. And from the decrease in TG content, it can be known that dioscin exerted a regulatory effect on lipid metabolism.

### 2.2. DIO Alleviates Kidney Damage in Rats

**Figure 2:**
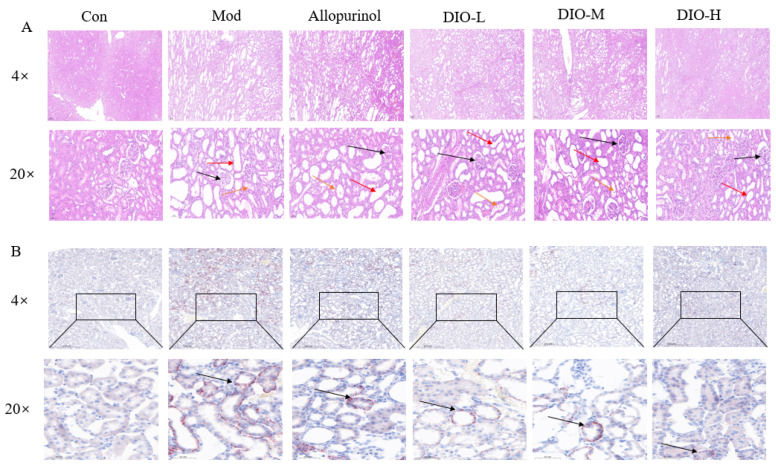
Effects of DIO on renal histopathology in model rats. (**A**) Hematoxylin–eosin (H&E) staining of kidney (original magnification ×4, scale bar 200 μm; original magnification ×20, scale bar 50 μm). (**B**) Oil red O staining of renal tissues (original magnification ×4, scale bar 200 μm; original magnification ×20, scale bar 50 μm). black arrows in panel (**A**) indicate enlarged glomerular capsule lumen and glomerular atrophy; red arrows indicate enlarged tubular lumen; yellow arrows indicate detachment of the brush border within the proximal tubule. In panel (**B**), the black arrows indicate that the red substances represent lipid droplets. Con: control group; Mod: model group; Allopurino: allopurinol group; DIO-L, M, H: Dio low-, medium-, and high-dose groups.

In order to explore the kidney-protective effect of dioscin on model rats, HE staining was performed on renal tissues. The results showed that the renal tissue structure was normal in the Con group; in the Mod group, glomerular atrophy, tubular dilation, and vacuolar degeneration of renal tubular epithelial cells were observed; after treatment with dioscin, the kidney damage was improved to some extent, and the effect was most obvious in the high-dose group (Figure 2A).

### 2.3. Dio Attenuates Lipid Accumulation in Rat Kidney

To investigate the lipid accumulation in the kidneys of model rats and the effect of dioscin, oil red O staining was performed on renal tissues. The results showed that the renal tissues in the Con group were normal without obvious red lipid droplets. In the Mod group, obvious red lipid droplets appeared in the renal tubules. The improvement effect in the positive drug group was not significant, while the number of red lipid droplets in the low-, medium- and high-dose groups of dioscin decreased significantly (Figure 2B), indicating that dioscin can alleviate the lipid accumulation in the kidneys of rats in the model group.

### 2.4. Dioscin Improves the Levels of Inflammatory Markers in the Renal Tissues of Model Rats and Has No Significant Impact on the Activity of Xanthine Oxidase (XO) in the Liver

**Figure 3:**
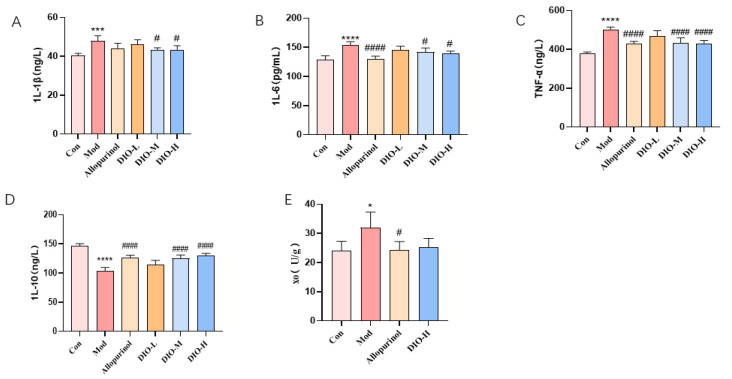
Effect of diosgenin on renal inflammatory factors and xanthine oxidase activity. (**A**) 1L-1β. (**B**) 1L-6. (**C**) TNF-α. (**D**) 1L-10. (**E**) Xanthine oxidase activity. **** *p* < 0.0001, *** *p* < 0.001, * *p* < 0.05 vs. control group; ^####^ *p* < 0.0001, ^#^ *p* < 0.05 vs. model group. Data are expressed as mean ± standard deviation (SD), *n* = 5. Con: control group; Mod: model group; Allopurino: allopurinol group; DIO-L, M, H: DIO low-, medium-, and high-dose groups.

To evaluate the inflammatory markers in renal tissues, the results showed that compared with the Con group, the levels of pro-inflammatory factors such as IL-1β, IL-6, and TNF-α in the kidneys of the Mod group were significantly increased (*p* < 0.0001, *p* < 0.001; Figure 3A–C), while the level of the anti-inflammatory factor IL-10 was significantly decreased (*p* < 0.0001; Figure 3D). In the medium- and high-dose groups of Dioscin, the levels of pro-inflammatory factors IL-1β, IL-6, and TNF-α were significantly decreased (*p* < 0.0001, *p* < 0.05; Figure 3A–C), and the levels of the anti-inflammatory factor IL-10 in the medium- and high-dose groups were significantly increased (*p* < 0.0001; Figure 3D).

The results of detecting the activity of xanthine oxidase (XO) in the liver showed that compared with the Con group, the activity of xanthine oxidase in the Mod group was significantly increased (*p* < 0.05; Figure 3E), but there was no significant difference between the high-dose group and the model group. These findings indicate that Dioscin alleviates renal injury in model rats by regulating inflammatory markers. Studies have found that reducing uric acid in the body generally involves reducing uric acid production by inhibiting the activity of liver XO or increasing uric acid excretion by the kidneys. However, the results of XO activity suggest that Dioscin does not affect the production of uric acid by influencing the activity of liver XO enzyme to reduce the uric acid level in model rats.

### 2.5. Transcriptomics Analysis of Renal Tissues and Verification of Differential Genes

**Figure 4:**
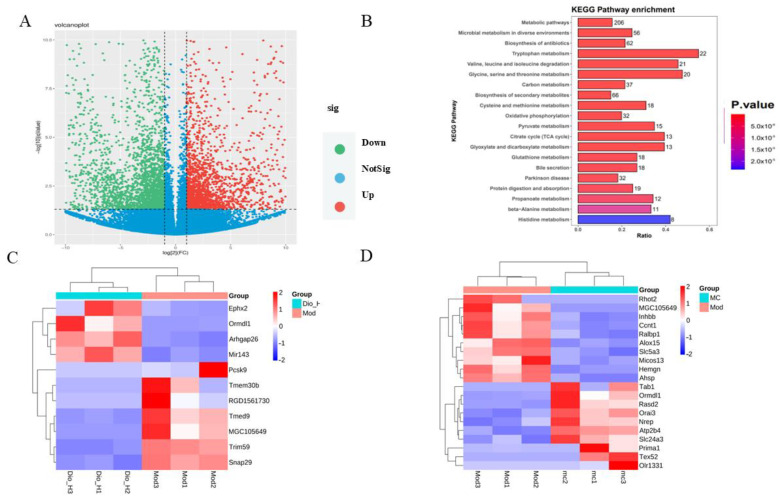
Transcriptomics results. (**A**) Volcano plot of DEGs based on the criteria of |log2(FC)| > 1 and *p*-value < 0.05. (**B**) KEGG pathways with the top 20 enrichment degrees. (**C**) Differential gene expression in DIO group vs. model group. (**D**) Differential gene expression between model group and model control group.

**Figure 5:**
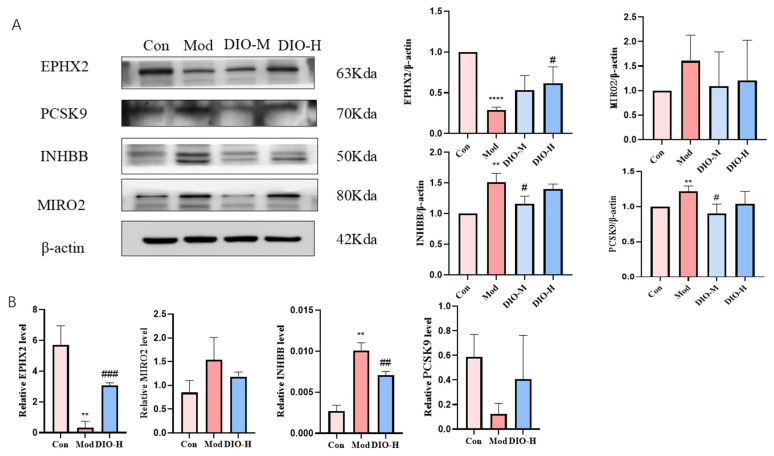
Differential gene validation results. (**A**) Differential gene Western blotting results. (**B**) Differential gene PCR results. **** *p* < 0.0001, ** *p* < 0.01 vs. control group; ^###^ *p* < 0.001, ^##^ *p* < 0.01, ^#^ *p* < 0.05 vs. model group. The data were expressed as the mean ± standard deviation (SD), *n* = 3. Con: control group; Mod: model group; DIO-M, DIO-H: DIO medium- and high-dose groups.

To explore the protective mechanism of Dioscin on model rats with plateau hyperuricemia, transcriptomics analysis was conducted on renal tissues. A total of 3586 differentially expressed genes (DEGs) (|fold change| > 2, *p*-value < 0.05) were identified through transcriptome sequencing between the control group and the model group, among which 1611 genes were upregulated and 21,975 genes were downregulated (Figure 4A). A total of 847 DEGs were detected through sequencing between the model group and the high-dose Dioscin group, with 403 genes being upregulated and 444 genes being downregulated. Furthermore, KEGG pathway enrichment analysis was carried out on the potential targets of DEGs. The 3586 DEGs between the control group and the model group were enriched in a total of 314 enrichment pathways (Figure 4B). The differential genes were mainly significantly enriched in pathways related to metabolic pathways. According to the pathways in which the differential genes were enriched, four DEGs related to metabolic pathways, namely EPHX2, PCSK9, MIRO2, and INHBB, were selected from the top 20 genes with the highest enrichment degrees of differential genes among the control group and the model group, the high-dose Dioscin group, and the model group, and the model control group and the model group. After verification and selection through Western blotting (Figure 5A) and RT-PCR (the primer sequences are shown in Table 1) (Figure 5B), the results showed that the protein expression of EPHX2 was downregulated in the model group and upregulated in the high-dose group (*p* < 0.01, *p* < 0.001; Figure 5A). The gene expression of EPHX2 was downregulated in the model group and upregulated in the high-dose group (*p* < 0.0001, *p* < 0.05; Figure 5B, which was consistent with the transcriptomics results. However, there were no significant differences in either the protein expression or the gene expression of MIRO2 (Figure 5A,B). The protein expression of INHBB was upregulated in the model group and downregulated in the high-dose group (*p* < 0.01; Figure 5A), while its gene expression was upregulated in the model group, downregulated in the medium-dose group, and showed no significant difference in the high-dose group (*p* < 0.01, *p* < 0.05; Figure 5B). The protein expression of PCSK9 was downregulated in the model group and upregulated in the high-dose group, but without significant differences (Figure 5A). Its gene expression was upregulated in the model group, downregulated in the medium-dose group, and showed no significant difference in the high-dose group (*p* < 0.01, *p* < 0.05; Figure 5B), indicating that the results of protein expression and gene expression were inconsistent. Since only the results of EPHX2 in transcriptomics, Western blotting, and RT-PCR were consistent, the EPHX2 gene was selected for subsequent studies.

### 2.6. Establishment of a Renal Tubular Epithelial Cell (HK-2) Injury Model for Plateau Hyperuricemia and Screening of Concentrations of Diosgenin (DG) and EPHX2 Inhibitors

**Figure 6:**
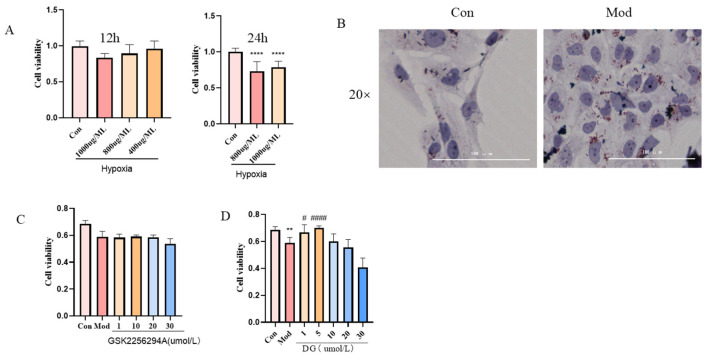
Modeling of renal tubular epithelial cell injury in plateau hyperuricemia. (**A**) Cell model screening conditions. (**B**) Lipid accumulation in model cells. (**C**) Diosgenin administration concentration screening. (**D**) Concentration screening of EPHX2 inhibitor GSK2256294A. **** *p* < 0.0001, ** *p* < 0.01 vs. control group; ^####^ *p* < 0.0001, ^#^ *p* < 0.05 vs. model group. Data are expressed as mean ± standard deviation (SD), *n* = 3. Con: control group; Mod: model group; DG: diosgenin group; GSK2256294A: EPHX2 inhibitor.

A cell model was established by culturing with 1 mg/mL uric acid combined with 1% O_2_ hypoxia for 24 h. The results showed that the cell viability was significantly decreased (*p* < 0.0001; Figure 6A). After oil red O staining, obvious red lipid droplets appeared in the model group (Figure 6B). The results were consistent with those of the animal model, indicating that the renal tubular epithelial cell injury model for plateau hyperuricemia was successfully established. An amount of 5 μmol/L of Diosgenin (DG) could significantly increase cell viability (*p* < 0.0001; Figure 6C). Different concentrations of GSK2256294A did not significantly reduce cell viability. Therefore, 5 μmol/L DG and 20 μmol/L GSK2256294A were selected for subsequent experiments.

### 2.7. DG Ameliorates Lipid Accumulation in the HK-2 Cell Model Through EPHX2

**Figure 7:**
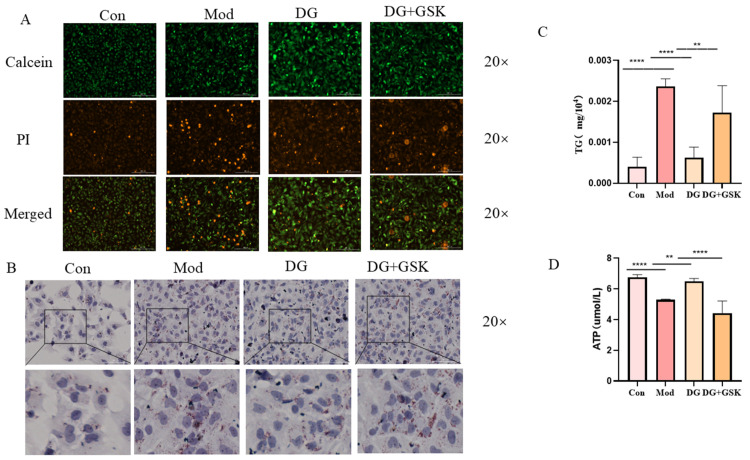
DG attenuates HK-2 cell damage (**A**) Results of HK-2 cell live color staining. (**B**) HK-2 cell oil red O staining results. (**C**) Triglyceride assay. (**D**) ATP assay. Compared with each group, **** *p* < 0.0001, ** *p* < 0.01. Data are expressed as mean ± standard deviation (SD), *n* = 5. Con: control group; Mod: model group; DG: diosgenin group; DG + GSK: inhibitor group.

To evaluate the therapeutic effect of DG on the HK-2 cell model, after cell modeling and drug administration, cell viability and death staining were performed. The results showed that compared with the control group, the number of dead cells in the model group increased (Figure 7A). Compared with the model group, the DG group could reduce the number of dead cells (Figure 7A). Compared with the DG group, the number of dead cells increased in the DG + GSK group (Figure 7A). To evaluate the improvement effect of DG on lipid accumulation in the HK-2 cell model, oil red O staining and triglyceride (TG) content detection were carried out. The results indicated that compared with the control group, the number of lipid droplets in the model group increased (Figure 7B), and the TG content was significantly elevated (*p* < 0.0001; Figure 7C). Compared with the model group, the number of lipid droplets in the DG group decreased (Figure 7B), and the TG content was significantly reduced (*p* < 0.0001; Figure 7C). Compared with the DG group, the number of lipid droplets in the DG + GSK group increased (Figure 7B), and the TG content was significantly increased (*p* < 0.01; Figure 7C). The kidneys generally rely on fatty acid metabolism for energy supply. Disordered lipid metabolism will affect the production of ATP in the kidneys. The experimental results demonstrated that compared with the control group, the ATP content in the model group was significantly decreased (*p* < 0.0001; Figure 7D). Compared with the model group, the ATP content in the DG group was significantly increased (*p* < 0.01; Figure 7D). Compared with the DG group, the ATP content in the DG + GSK group was significantly decreased (*p* < 0.0001; Figure 7D). Overall, DG may regulate lipid metabolism in the HK-2 cell model, reduce lipid accumulation, and alleviate cell death. This regulatory effect may be achieved by upregulating the EPHX2 gene.

### 2.8. DG Ameliorates Oxidative Stress and Inflammatory Injury in the HK-2 Cell Model

**Figure 8:**
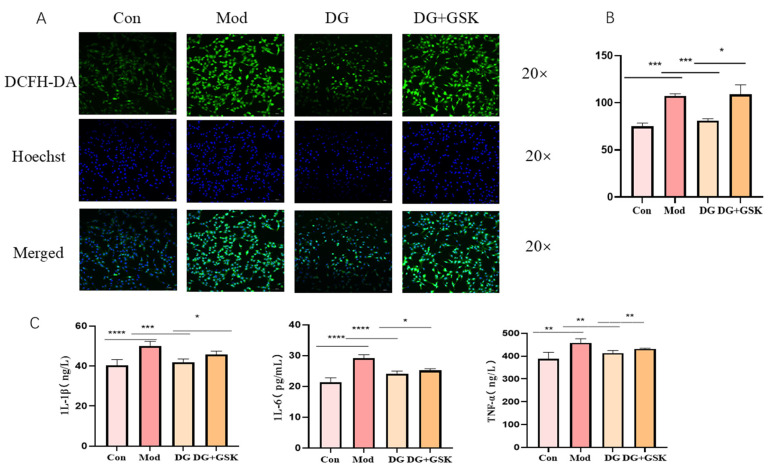
Diosgenin attenuates ROS and inflammatory factors in HK-2 cells (**A**) ROS staining results of HK-2 cells. (**B**) ROS fluorescence statistic graph. (**C**) Results of 1L-6, 1L-1β, and TNF-α assays on HK-2 cell supernatants. Compared with each group, **** *p* < 0.0001, *** *p* < 0.001, ** *p* < 0.01, * *p* < 0.05, Data are expressed as mean ± standard deviation (SD), *n* = 5. Con: control group; Mod: model group; DG: diosgenin group; DG + GSK: inhibitor group.

Lipid accumulation can cause oxidative stress and inflammatory responses in cells. To evaluate the oxidative stress and inflammatory injury of the HK-2 cell model, ROS staining and the detection of inflammatory factors were conducted after cell modeling and drug administration. The results showed that compared with the control group, the level of ROS in the model group was significantly increased (*p* < 0.001; Figure 8A,B). Compared with the model group, the level of ROS in the DG group was significantly decreased (*p* < 0.001; Figure 8A,B). Compared with the DG group, the level of ROS in the DG + GSK group was significantly increased (*p* < 0.05; Figure 8A,B). The results of the detection of inflammatory factors indicated that compared with the control group, the levels of IL-1β, IL-6, and TNF-α in the model group were all significantly increased (*p* < 0.0001, *p* < 0.01; Figure 8C). Compared with the model group, these levels in the DG group were all significantly decreased (*p* < 0.0001, *p* < 0.01; Figure 8C). Compared with the DG group, these levels in the DG + GSK group were significantly increased (*p* < 0.05, *p* < 0.01; Figure 8C). Overall, DG may reduce lipid accumulation in HK-2 model cells by upregulating the EPHX2 gene and thereby alleviate oxidative stress and inflammatory injury in HK-2 model cells.

### 2.9. EPHX2 Possible Successor Pathway Genes

**Figure 9:**
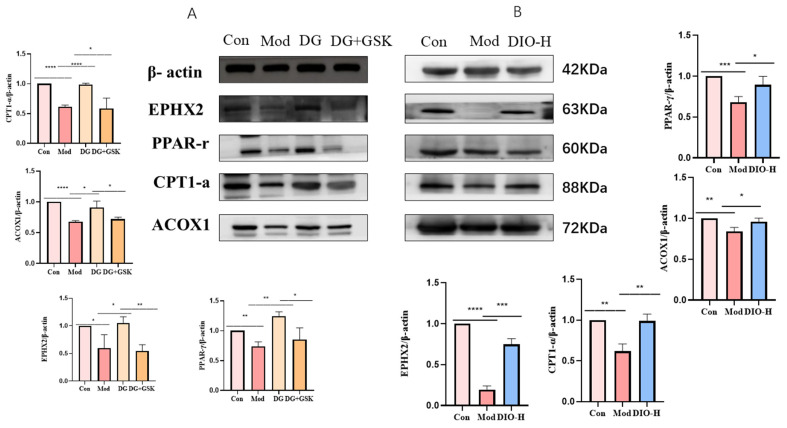
Validation results of target genes and possible downstream genes. (**A**) Cell Western blotting results (**B**) Kidney tissue Western blotting results. Compared with each group, **** *p* < 0.0001, *** *p* < 0.001, ** *p* < 0.01, * *p* < 0.05; data are expressed as mean ± standard deviation (SD), *n* = 3. Con: control group; Mod: model group; DG: diosgenin meta-group; DG + GS: inhibitor group; DIO-H: dioscin high dose group.

The EPHX2 gene encodes epoxide hydrolase (soluble epoxide hydrolase, SEH). SEH is a bifunctional enzyme. The C-terminal domain has epoxide hydrolase activity and can convert epoxides into corresponding diols, while the N-terminal domain has phosphatase activity for phospholipid hydrolysis [15,16]. Studies have found that the EPHX2 gene can inhibit the development of colorectal cancer by inhibiting lipid metabolism [15]. Knocking out the EPHX2 gene in mice will affect their basic glycolipid metabolism [16]. To identify which specific link in lipid metabolism is regulated by the EPHX2 gene, Western blotting was used to detect Acyl Coenzyme A oxidase (ACOX) and Carnitine palmitoyltransferase (CPT) in the process of lipid metabolism. The enzyme ACOX is mainly responsible for oxidizing long-chain fatty acids into medium- and short-chain fatty acids, while CPT is responsible for transporting short-chain fatty acids into the mitochondria of renal tubular epithelial cells for β-oxidation [4]. The results showed that compared with the control group, the expression of EPHX2 in the cell model was downregulated (*p* < 0.05; Figure 9A), and both ACOX1 and CPT1α were also downregulated (*p* < 0.0001; Figure 9A). Compared with the model group, the expression of EPHX2 in the DG group was upregulated (*p* < 0.05; Figure 9A), and both ACOX1 and CPT1α were upregulated (*p* < 0.05, *p* < 0.0001; Figure 9A). Compared with the DG group, the expression of EPHX2 in the DG + GSK group was significantly downregulated (*p* < 0.01; Figure 9A), and both ACOX1 and CPT1α were also significantly downregulated (*p* < 0.05; Figure 9A). The results of the Western blotting of rat kidney tissues showed that compared with the control group, the EPHX2 gene in the model group was significantly downregulated (*p* < 0.0001; Figure 9B), and both ACOX1 and CPT1α were downregulated (*p* < 0.01; Figure 9B). Compared with the model group, the expression of EPHX2 in the DIO group was significantly upregulated (*p* < 0.001; Figure 9B), and both ACOX1 and CPT1α were upregulated (*p* < 0.05, *p* < 0.01; Figure 9B).

The peroxisome proliferator-activated receptor (PPAR) is a family of nuclear hormone receptors. Among them, PPARγ is a key regulator of renal lipid metabolism [17]. Studies have found that the activation of PPARγ is related to the expression of sEH in human endothelial cells [18]. The results of Western blotting detection showed that compared with the control group, the expression of EPHX2 in the cell model was downregulated (*p* < 0.05; Figure 9A), and PPAR-γ was also downregulated (*p* < 0.01; Figure 9A). Compared with the model group, the expression of EPHX2 in the DG group was upregulated (*p* < 0.05; Figure 9A), and PPAR-γ was upregulated (*p* < 0.01; Figure 9A). Compared with the DG group, the expression of EPHX2 in the DG + GSK group was significantly downregulated (*p* < 0.01; Figure 9A), and PPAR-γ was also significantly downregulated (*p* < 0.05; Figure 9A). The results of Western blotting of rat kidney tissues showed that compared with the control group, the EPHX2 gene in the model group was significantly downregulated (*p* < 0.0001; Figure 9B), and PPAR-γ was downregulated (*p* < 0.001; Figure 9B). Compared with the model group, the expression of EPHX2 in the DIO group was significantly upregulated (*p* < 0.001; Figure 9B), and PPAR-γ was upregulated (*p* < 0.05; Figure 9B).

Overall, the EPHX2 gene may have an impact on lipid metabolism by regulating the genes related to lipid metabolism, such as ACOX1, CPT1α, and PPAR-γ.

## 3. Discussion

Affected by the hypoxic environment on the plateau, hyperuricemia has become one of the common diseases in plateau areas. Studies have found that kidney damage caused by plateau hypoxia, which leads to reduced uric acid excretion, is an important predisposing factor for the high incidence of hyperuricemia. Lipid accumulation caused by the hypoxic environment can also trigger oxidative stress and inflammatory responses in the kidneys, further causing kidney damage, reducing the kidneys’ excretion of uric acid, and thus inducing hyperuricemia [1]. Therefore, it is of great significance to explore the role of kidney lipid metabolism disorders in causing kidney oxidative stress and inflammatory responses for the treatment of plateau hyperuricemia. Dioscin has been widely studied in aspects such as reducing uric acid, anti-inflammation, and anti-tumor [7,8,9]. Both DIO and its active metabolite DG can alleviate kidney oxidative stress and inflammatory responses through multiple pathways [11,12,13,14].

In this study, plateau hyperuricemia was first established by combining PO and HPX with hypobaric hypoxia. After treatment with Dio, the levels of UA, CREA, and BUN in the model rats were effectively reduced, demonstrating a significant effect on reducing uric acid. Generally, reducing the level of uric acid in the body is achieved by reducing uric acid production or increasing its excretion. Liver xanthine oxidase is a key enzyme in uric acid production [19]. However, the experimental results showed that DIO could not effectively reduce the activity of liver XO. This indicates that DIO reduces the level of uric acid in the body by increasing kidney excretion. Kidney index, CREA, and BUN are generally regarded as key indicators for the integrity of kidney function [20]. The kidney index, CREA, and BUN of the model rats all increased significantly. In addition, histopathological examination of the kidneys of the model rats showed glomerular atrophy, tubular dilation, and vacuolar degeneration of renal tubular epithelial cells, indicating that the kidneys of the model rats were damaged. After treatment with dioscin, the kidney index, CREA, and BUN all decreased significantly, suggesting that kidney function was improved. Histopathological examination further confirmed this improvement, as glomerular damage in DIO-treated rats was alleviated and the degree of tubular dilation was also reduced. Some studies have shown that the kidneys mainly rely on fatty acid metabolism for energy supply, and plateau hypoxia can cause lipid metabolism disorders in the kidneys of rats, resulting in lipid accumulation. Therefore, oil red O staining was performed on the kidneys of the model rats. The results showed that a large number of red lipid droplets appeared in the rat kidneys, mostly concentrated around the renal tubules. The renal tubules are mainly responsible for reabsorption, secretion, and excretion, and the kidneys’ excretion of uric acid mainly depends on the renal tubules [21]. Renal tubular epithelial cells, as one of the most energy-consuming cells in the body, are also the most sensitive to hypoxia [4]. The results of oil red O staining of rat kidneys showed that after treatment with Dio, the red lipid droplets accumulated around the renal tubules were significantly reduced. Excessive lipid accumulation in the kidneys may lead to lipotoxicity in the kidneys, causing oxidative stress and inflammatory damage to kidney tissues [6]. The results of detecting inflammatory factors in the kidneys of the model rats showed that the contents of IL-6, IL-1β, and TNF-α increased, while the content of IL-10 decreased. After treatment with Dio, the contents of IL-6, IL-1β, and TNF-α were significantly reduced, and the content of IL-10 was significantly increased, indicating that Dio may reduce lipid accumulation in the kidneys, alleviate the inflammatory response, protect the kidneys, and increase their excretion of uric acid. It should be emphasized that due to the limited number of rats that the hypobaric oxygen chamber can accommodate, only five animals were selected for each group to conduct the experiment. Considering the natural differences among individual organisms, this has, to some extent, restricted the comprehensiveness of the research. However, in order to ensure the reproducibility of the experimental data, subsequent replicate experiments were carried out under the same conditions, and the results of the two experiments were consistent.

Transcriptome sequencing can comprehensively and rapidly obtain the sequence information and expression information of almost all transcripts in specific cells or tissues under a certain state, thus accurately analyzing differences in gene expression. In this study, transcriptomics was used to explore the mechanism of kidney damage in model rats with plateau hyperuricemia and the mechanism by which Dio exerts its therapeutic effect. The results showed that both were closely related to lipid metabolism. Finally, based on gene enrichment degree screening and verification through Western blotting and RT-PCR experiments, the EPHX2 gene was selected as the subject for subsequent research. Studies have found that EPHX2 can not only participate in lipid metabolism but also regulate vascular pressure and inflammatory responses by regulating lipid metabolism [15,16]. To explore the mechanism by which Dioscin regulates kidney lipid metabolism in model rats through EPHX2, human renal cortical proximal tubule epithelial cells (HK-2) were selected for cell modeling. After culturing with 1 mg/mL uric acid combined with 1% O_2_ hypoxia for 24 h, the cells showed reduced viability and lipid accumulation, and the plateau hyperuricemic renal tubular epithelial cell injury model was successfully established. However, after treatment with DIO, the lipid accumulation situation could not be improved. Studies have found that DIO will be metabolized into DG with higher lipid solubility in vivo [13]. After DG was administered, the lipid accumulation in the cells was significantly improved (see attached figures). After successful cell modeling, the cells were divided into the control group, model group, DG group, and DG + GSK group for experiments after drug administration. The results showed that in the DG group, the number of dead cells decreased, the TG (triglyceride) content decreased, lipid accumulation was alleviated, and the contents of ROS, IL-6, IL-1β, and TNF-α were all reduced. In the DG + GSK group with the addition of the EPHX2 inhibitor, the number of dead cells increased, the TG content increased, lipid accumulation was aggravated, and the contents of ROS, IL-6, IL-1β, and TNF-α increased. This indicates that DG exerts its role in regulating lipid metabolism and alleviating oxidative stress and inflammatory responses in model cells through the EPHX2 gene. Fatty acid metabolism involves multiple metabolic enzymes, among which the more important ones are acyl-CoA oxidase (ACOX), carnitine palmitoyltransferase (CPT), and peroxisome proliferator-activated receptor γ (PPARγ). Studies have found that after DIO upregulates EPHX2, ACOX1, CPT1α, and PPARγ are all upregulated, while after inhibiting EPHX2, ACOX1, CPT1α, and PPARγ are all downregulated. The results indicate that EPHX2 plays its role by regulating multiple aspects of fatty acid metabolism.

The research results suggest that the therapeutic effect of dioscin on plateau hyperuricemia may be achieved by its active metabolite diosgenin regulating the EPHX2 gene to regulate kidney lipid metabolism, alleviating kidney lipid accumulation, thereby reducing kidney inflammatory damage, producing a kidney-protective effect, and increasing the kidneys’ excretion of uric acid.

## 4. Materials and Methods

### 4.1. Animals, Reagents, and Instruments

Thirty SPF male Sprague Dawley rats (7–8 weeks, 300–320 g) were purchased from Beijing Viton Lihua Laboratory Animal Technology Co., Ltd. (certification no.: SCXK (Beijing) 2021-0011, Beijing, China), The rats were housed under standard conditions: 12 h light/dark cycle, controlled temperature of 20–25 °C, and a relative humidity of (50 ± 5)%. The experiment was conducted in the central laboratory of Animal Ethics Committee of the Institute of Military Medical Research (Beijing, China). All animal studies were conducted following the Animal Ethics Committee of the Institute of Military Medical Research (approval No. IACUC-DWZX-2024-542).

HK-2 cells (Wuhan Punosai Life Science and Technology Co., Ltd., cat. no. CL-0109, Wuhan, China) were cultured in complete DMEM/F12 medium (Gibco, cat. no. C11330500BT, SuZhou, China) supplemented with 10% fetal bovine serum (Zhejiang Tianhang Biological, cat. no. 11011-8611, ZheJiang, China) and 1% penicillin-streptomycin (Wuhan Punosai Life Science and Technology Co., Ltd., cat. no. PB180120, Wuhan, China). The cells were incubated at 37 °C with 5% CO_2,_ and the medium was changed every three days. When the cell density reached 80–90% confluence, the experiment was initiated by replacing the complete medium with induction medium (incomplete medium containing 1 mg/mL uric acid).

Dioscin (purity > 98%, cat. no. N09IB231464, Shanghai, China), Potassium oxonate (PO) (purity > 98%, cat. no. S17112 Shanghai, China), and Hypoxanthine (HPX) (purity > 98%, cat. no. B20211 Shanghai, China) were all purchased from Shanghai Yuanye Bio-Technology Co., Ltd.; Diosgenin (cat. no. HY-N0177, NJ, USA) and GSK2256294A (GSK) (cat. no. HY-19644, Warren, NJ, USA) were purchased from Med Chem Express (Princeton, NJ, USA). Sodium carboxymethyl cellulose (CMC-Na) (cat. no. CP300, Shanghai, China) was purchased from Sinopharm Chemical Reagent Co. Allopurinol (cat. no. A21418, Beijing, China) and dimethyl sulfoxide (DMSO) (cat. no. D3855, Beijing, China) was purchased from Beijing InnoChem Science & Technology Co., Ltd. MIRO2 Polyclonal antibody (MIRO2, cat. no.11237-1-Ap, WuHan, China), PCSK9 Polyclonal antibody (PCSK9, cat. no. 27882-1-Ap, Wuhan, China), EPHX2 Polyclonal antibody (EPHX2, cat. no.10833-1-Ap, Wuhan, China), ANGPTL4 Polyclonal antibody (ANGPTL4, cat. no.18374-1-Ap, Wuhan, China), and CPT1A Rabbit PolyAb (CPT1A, cat. no. 15184-1-Ap Wuhan, China) were purchased from ProteinTech. Inhibin beta B Rabbit mAb (INHBB, cat. no. R389280, Chengdu, China), ACOX1 Rabbit mAb (ACOX1, cat. no. R23371, Chengdu, China), Phospho-PPAR gamma (Thr166) Rabbit pAb (PPAR-γ, cat. no. 370374, Chengdu, China), and Goat Anti-Rabbit IgG H&L (cat. no. 550048, Chengdu, China) were purchased from Chengdu Zen Bioscience Co., Ltd.; PPAR alpha Antibody (PPAR-α, cat. no. AF5301, Melb, Australia) was purchased from Affinity Biosciences; uric acid (cat. no. U105582, Shanghai, China) was purchased from Aladdin. 

### 4.2. Experimental Design

**Figure 10:**
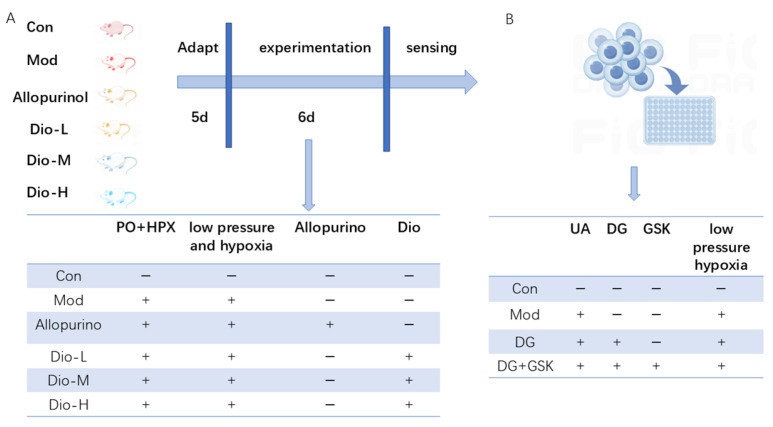
Experimental flowcharts. (**A**) Flowchart of animal experiments. (**B**) Flowchart of cell experiments.

A rat model of plateau hyperuricemia was established by administering modeling drugs (500 mg/kg PO + 500 mg/kg HPX) combined with hypobaric hypoxia. After one week of adaptive feeding, 30 Sprague Dawley (SD) rats were randomly divided into 6 groups, with 5 rats in each group. Except for the control group, the modeling drugs were intragastrically administered according to the body mass of rats. One hour later, different doses of DIO were intragastrically administered to the treatment groups, and 10 mg/kg of allopurinol was intragastrically administered to the positive drug group. Then, the model group, DIO group, and allopurinol group were placed in a hypobaric oxygen chamber for 6 consecutive days (Figure 10A).

HK-2 cell model was established by using induction medium (incomplete medium containing 1 mg/mL uric acid) to culture the cells and put them into 1% O_2_ concentration hypoxia incubator for 24 h, the dosing group was added with the appropriate dose of DG, and the inhibitor group was added with the same concentration of DG as that of the dosing group and the appropriate concentration of EPHX2 inhibitor (GSK2256294A), which was dissolved in the induction medium. Anoxic incubation was performed for 24 h (Figure 10B).

### 4.3. Serum Collection and Biochemical Analysis

After the final administration, the rats were fasted for 24 h. They were then anesthetized with 2% pentobarbital (3 mL/kg). Blood was collected from the abdominal aorta and centrifuged at 3000 rpm for 15 min to obtain serum. Kidney tissues were also collected; a portion of the kidneys was fixed in 10% formalin for histological examination, while the remaining kidney tissue was snap-frozen in liquid nitrogen and stored at −80 °C for subsequent analyses. Serum levels of uric acid (UA), creatinine (CREA), blood urea nitrogen (BUN), and triglycerides (TG) were measured using a fully automated biochemical analyzer (Cobas C311, Roche, CA, USA).

### 4.4. Histopathological Analysis

The sections were stained with hematoxylin–eosin staining (HE) and observed morphologically under a light microscope (NIKON ECLIPSE, NIKON, Tokyo, Japan).

### 4.5. Oil Red O Staining of Kidney

Oil red O staining was performed on the sections, and renal lipids were visualized under a light microscope (NIKON ECLIPSE, NIKON, Tokyo, Japan).

### 4.6. Inflammatory Factors and Xanthine Oxidase Enzyme Activity Assay

Enzyme-linked immunosorbent assay (ELISA) kit was used to detect interleukin-6 (cat. no. MM-0180R2, Enzyme Free Biotechnology Co., Ltd., Nanjing, China), interleukin-1β (cat. no. MM-0047R2, Enzyme Free Biotechnology Co., Ltd., Nanjing, China), Tumor Necrosis Factor-α (cat. no. MM-0180R2, Enzyme Immunity Biotechnology Co., Ltd., Nanjing, China), and xanthine oxidase activity (cat. no. BC1095, Solebo, Beijing, China). These assays were performed according to the manufacturer’s instructions to quantify the levels of IL-6, IL-1β, TNF-α, and xanthine oxidase activity in the samples.

### 4.7. Kidney Transcriptomics

Kidney samples from the control, model, DIO-H, and model control groups (3 samples from each group) were frozen in liquid nitrogen and stored at −80 °C, and then sent to Beijing Qinglian Bio Co. (Beijing, China). Raw data were filtered to obtain high-quality data (Clean Data), and HISAT2 was used to compare the Clean Data with the designated reference genome, calculate the efficiency of comparison between the sequencing data and the reference genome, and evaluate the saturation of the sequencing data and the coverage of the genes; based on the results of the comparison, transcript assembly was carried out, and the expression amounts of genes were calculated in different samples to construct the gene expression profiles. Based on the comparison results, transcript assembly was performed to calculate the gene expression in different samples and construct gene expression profiles. Gene expression profiles were constructed by using the FPKM method to normalize the gene expression in the samples, and the R toolkit DESeq2 was used to analyze the differentially expressed genes (DEGs) among the groups, and the genes that meet the criteria of |log2 (fold change)| > 1 are defined as differentially expressed genes. Subsequently, the above results were enriched and annotated using the Kyoto Encyclopedia of Genes and Genomes (KEGG, http://www.genome.jp/kegg/pathway.html) (accessed on 8 January 2024) and GO databases to analyze the entries that were significantly enriched in the DEGs list.

### 4.8. Total Protein Extraction and Western Blot Analysis

Total protein extraction from renal tissues was performed using radioimmunoprecipitation assay (RIPA) buffer (cat. no. PC102, Yase Biologicals, Shanghai, China). The extracted protein samples were mixed with one-fourth volume of 5× loading buffer and denatured at 100 °C for 10 min. The samples were then subjected to 10% sodium dodecyl sulfate-polyacrylamide gel electrophoresis (SDS-PAGE) for 90 min. After electrophoresis, the proteins were transferred to a polyvinylidene fluoride (PVDF) membrane. The membrane was blocked with 5% skimmed milk (cat. no. PS112L, Yase Biologicals, Shanghai, China) for 60 min at room temperature. Subsequently, the membrane was incubated with the following primary antibodies overnight at 4 °C: rabbit anti-PCSK9, EPHX2, MIRO2, INHBB, β-actin, CPT1A, ACOX1, PPARα, and PPARγ (all antibodies at 1:1000 dilution). The membrane was then washed with TBST and incubated with horseradish peroxidase (HRP)-conjugated goat anti-rabbit secondary antibody (1:5000 dilution) for 1 h at room temperature. After washing, the blots were visualized using an enhanced chemiluminescence (ECL) detection system on an electrophoresis gel imager (LA3500, ImageQuant, Marlborough, MA, USA). The band intensities were analyzed using ImageJ software (ImageJ 1.54g, National Institutes of Health, Bethesda, MD, USA), and the protein expression levels were normalized to the internal reference gene (β-actin).

### 4.9. Renal PCR for Verification of Differential Genes Total RNA Extraction and Reverse Transcription

Total RNA was extracted with TRIzol (cat. no.BCCK3647, Sigma, St. Louis, MO, USA), and reverse transcription was performed with the Reverse Transcription Kit (cat. no. 11123ES60, Next Sage Bio, Shanghai, China). Each qRT-PCR consisted of 2 ug of RNA, 1 μL of DNA digester, and 2 μL of 5× DNA digester buffer. The mixture was incubated for 2 min at 42 °C before adding RNase-free H20 until it reached 10 μL; it was then incubated at 42 °C for 2 min before adding 2× Hifair II Super Mix plus 10 μL. qRT-PCR cycles were 25 °C for 5 min, 42 °C for 30 min, and 85 °C for 5 min. Then, using the cDNA sample as a template, 0.4 μL of forward and reverse primers, 10 μL of Hieff qPCR SYBR Green Master Mix, and 20 μL RNase-free H20 were added, and the real-time qPCR cycle was set to 95 °C for 5 min and 95 °C for 10 s, 55 °C for 20 s, and 72 °C for 20 s. The qPCR cycle was repeated for 40 cycles. The data were analyzed by the 2^−ΔΔCT^ method by repeating 40 cycles (CFX Opus 96, BIO RAD, Hercules, CA, USA), and the primer sequences are tabulated in Table 1.

### 4.10. Cell Proliferation Assay (Diosgenin and EPHX2 Inhibitor Concentration Screening)

HK-2 cells were seeded in 96-well plates at a density of 10,000 cells per well and cultured for 12 h. After 12 h of culture, different concentrations of DG and the EPHX2 inhibitor (GSK2256294A) were dissolved in the induction medium and added to the 96-well plates. The plates were then incubated in a hypoxic incubator (Bugbox M, Baker Ruskinn, London, UK) at a 1% O_2_ concentration for 24 h. Following the 24 h incubation, 100 μL of CCK-8 reagent (diluted 1:10; cat. no. E1008, Beijing Pulilai, Beijing, China) was added to each well. The plates were incubated at 37 °C for 1 h. The absorbance values (OD) were measured at 450 nm using an enzyme-linked immunosorbent assay (ELISA) reader (VICTOR X5, PerkinElmer, Waltham, MA, USA).

### 4.11. Oil Red O Staining of HK-2 Cells

HK-2 cells were seeded in 6-well plates at a density of 100,000 cells/well and cultured for 12 h. Then, the cells were divided into control, model (induction medium), DG (5 umol/L dissolved in induction medium), and DG + GSK groups (5 umol/L DG + 20 umol/L GSK2256294A dissolved in induction medium), placed in a 1% O_2_ incubator for anoxic culture for 24 h, fixed with 4% paraformaldehyde (cat. no. B0006, Beijing Qinyi Biotech, Beijing, China) for 10 min, and washed twice with PBS. Then, staining wash solution was added to cover the cells for 20 s, and 1 mL of oil-red O staining workup (cat. no. C0157S, Beytime Biotech Inc., ShangHai, China) was added for 20 min. Then, they were washed twice with PBS, hematoxylin staining solution (cat. no. C0107, Beytime Biotech Inc., ShangHai, China) was added to re-stain the nucleus of the cells, washed with PBS for 20 s, and washed on a live cell imaging microplate detector (BioTek, Winooski, VT, USA) for observation.

### 4.12. Live-Dead Staining of HK-2 Cells

HK-2 cells were inoculated in 6-well plates at a density of 100,000 cells/well and cultured for 12 h. Then, the cells were divided into control group, model group (induction medium), DG group (5 umol/L dissolved in induction medium), and DG + GSK group (5 umol/L Diosgenin + 20 umol/L inhibitor dissolved in induction medium) and put into 1% O_2_ incubator. After performing anoxic incubation for 24 h, Calcein (cat. no.C002, DOJINDO, Kumamoto Prefecture, Japan) SPiDER-βGa (cat. no. SG02, DOJINDO, Kumamoto Prefecture, Japan) was added and stained for 10 min. The staining solution was discarded and PBS was added and observed on a live cell imaging microplate detector (Bio Tek, Winooski, VT, USA).

### 4.13. Determination of Cellular ATP Content

HK-2 cells were inoculated in 6-well plates at a density of 100,000 cells/well and cultured for 12 h. Then, the cells were divided into control group, model group (induction medium), DG group (5 umol/L dissolved in the induction medium) and DG + GSK group (5 umol/L diosgenin + 20 umol/L inhibitor dissolved in the induction medium) and put into 1% O_2_ incubator. After 24 h of anoxic culture, cells were collected and assayed according to the instructions of ATP assay kit (cat. no. S0026, Beytime Biotech Inc., ShangHai, China), and the samples were spiked and assayed on an enzyme labeling instrument (VICTORX5, PerkinElmer, Waltham, MA, USA).

### 4.14. Determination of Triglyceride (TG) Content

After completion of cell induction culture, cells were collected and assayed according to the instructions of the triglyceride assay kit (cat. no. KTB2200, Abbkine, Atlanta, GA, USA).

### 4.15. Cellular ROS Staining

HK-2 cells were inoculated at a density of 100,000 cells/well in 20 mm confocal Petri dishes. After the induction culture was completed, ROS stain (cat. no. S0033S, Beytime Biotech Inc., ShangHai, China) was added and incubated at 37 °C for 20 min, and the staining solution was removed to add Hoechst stain (cat. no. P0133, Beytime Biotech Inc., Jiangsu, China), and the staining solution was stained for 10 min and then removed. Staining solution and incomplete medium were observed under laser confocal microscope (Nikon, Tokyo, Japan).

### 4.16. Statistical Analysis

GraphPad Prism 9.0 software (GraphPad Software, Inc., San Diego, CA, USA) was used. Data were expressed as mean ± standard deviation (mean ± SD). Comparison of means between groups was performed by one-way ANOVA and LSD-*t* test. Differences were considered statistically significant at *p* < 0.05.

## 5. Conclusions

Our study demonstrated that dioscin can have a therapeutic effect on rats with plateau hyperuricemia by exerting a protective effect on the kidneys. After dioscin is metabolized into diosgenin in the body, it regulates rat renal lipid metabolism through the EPHX2 gene, reduces renal lipid accumulation, alleviates inflammatory reactions, and increases the excretion of uric acid by rat kidneys. These findings suggest that dioscin may be used to treat high altitude hyperuricemia.

## Figures and Tables

**Table 1:** Gene primer sequences.

Gene	Forward Primer (5′–3′)	Reverse Primer (5′–3′)
Β-actin	ACCCTAAGGCCAACCGTGAAAAG	CATGAGGTAGTCTGTCAGGT
PCSK9	GCACTGGAGAACCACACAGG	TGGCTGCATGACATTGCTTCTC
EPHX2	CGCGACACACAGCCTCGGCTTT	AGTCATGGCCAATGAACAC
MIRO2	ACTACCTGTGATGTCGCCTG	ATGGTAGCACACTGCACGAA
INHBB	GCAGACATCGCATCCGAAAA	AATGATCCAGTCGTTCCAGCC

## Data Availability

The data used to support the findings of this study are available from the corresponding author upon request.
